# Large-Scale Robot-Based Polymer and Composite Additive Manufacturing: Failure Modes and Thermal Simulation

**DOI:** 10.3390/polym14091731

**Published:** 2022-04-24

**Authors:** Saeed Akbari, Jan Johansson, Emil Johansson, Lenny Tönnäng, Seyed Hosseini

**Affiliations:** 1 RISE Research Institutes of Sweden, Box 104, SE-431 22 Mölndal, Sweden; jan.j.johansson@ri.se (J.J.); lenny.tonnang@ri.se (L.T.); seyed.hosseini@ri.se (S.H.); 2 Adaxis, 97 Allée Théodore Monod, 64210 Bidart, France; emil.johansson@adaxis.eu

**Keywords:** large-scale additive manufacturing, polymers and composites, failure modes, warpage and delamination, thermal simulation

## Abstract

Additive manufacturing (AM) of large-scale polymer and composite parts using robotic arms integrated with extruders has received significant attention in recent years. Despite the contributions of great technical progress and material development towards optimizing this manufacturing method, different failure modes observed in the final printed products have hindered its application in producing large engineering structures used in aerospace and automotive industries. We report failure modes in a variety of printed polymer and composite parts, including fuel tanks and car bumpers. Delamination and warpage observed in these parts originate mostly from thermal gradients and residual stresses accumulated during material deposition and cooling. Because printing large structures requires expensive resources, process simulation to recognize the possible failure modes can significantly lower the manufacturing cost. In this regard, accurate prediction of temperature distribution using thermal simulations is the first step. Finite element analysis (FEA) was used for process simulation of large-scale robotic AM. The important steps of the simulation are presented, and the challenges related to the modeling are recognized and discussed in detail. The numerical results showed reasonable agreement with the temperature data measured by an infrared camera. While in small-scale extrusion AM, the cooling time to the glassy state is less than 1 s, in large-scale AM, the cooling time is around two orders of magnitudes longer.

## 1. Introduction

Over the past decade, significant research effort has been devoted to the development of extrusion-based additive manufacturing (AM) of polymers and polymer matrix composites for different applications [1,2,3,4,5]. In this technique, a three-dimensional computer-aided design (CAD) model of the component is sliced into discrete layers to prescribe the location of material deposition. Pure polymer or short-fiber-reinforced polymer is then melted in an extruder and deposited on a build plate in a layer-by-layer manner to fabricate a complex three-dimensional geometry [6,7,8,9].

Large-scale material-extrusion AM of polymer and composite structures can vastly increase design freedom and flexibility in the production of structural components and hence reduce the material waste in production. However, incremental deposition of molten material layer-by-layer induces significant temperature gradients and poses challenges associated with internal thermal residual stresses and large deformations. As the deposited material cools it shrinks, and the mechanical constraint imposed by the previously deposited layers introduces significant residual stresses. This in turn results in damages such as warpage and delamination initiation and propagation across the layer boundaries. While the printed layer thickness is typically in the range of 0.1–0.3 in small-scale material-extrusion AM, in large-scale AM, the thickness can be up to 4.0 mm or more [10,11,12,13,14]. Thus, more time is needed for these thicker polymer layers to cool down. When the next layers are deposited, the previous layers may not be cooled down enough and may still be soft and flexible, and therefore cannot provide a rigid support for deposition of the next layers. As a result, deposited layers may buckle, collapse, or experience significant warpage. Addition of short fibers to the polymer matrix can enhance the stiffness of the printed structure and prevent warpage and large deformations, but it may weaken the bonding strength at the boundary of the layers and result in delamination. Here, we describe different failure mechanisms observed in large parts printed with a variety of polymers and composites.

Numerical simulation techniques can enable prediction of temperature and stress distribution within printed structures to optimize the process parameters of material-extrusion AM. Although there have been several studies on process simulation of material-extrusion AM [15,16,17,18,19,20,21,22], most of them focused on melt flow within the nozzle and ignored subsequent material deposition and solidification. For example, Serdeczny et al. [15] used computational fluid dynamics (CFD) simulations to analyze the polymer flow inside the hot end of the extruderduring material-extrusion AM. The purpose was to investigate the effects of nozzle diameter as well as those of the liquefier temperature and length on the melt flow characteristics. The model was able to capture the melting of an acrylonitrile butadiene styrene (ABS) filament and the recirculation region between the nozzle wall and the filament. In another study, Shadvar et al. [16] modeled the effect of melt flow rate and extruder temperature on die swelling, and found that a lower material flow rate and higher melt temperature decreased pressure drop inside the nozzle and also limited swelling of the extruded filament.

Brenken et al. [20] used finite element analysis (FEA) to simulate temperature and residual stress distribution in material-extrusion AM. They indirectly replicated the deposition process by employing progressive element activation, in which the initially inactive elements are activated following the position of the printing nozzle. Therefore, the melt flow was not modeled and the computational cost reduced significantly. The printed parts were merely represented by voxel elements, which had a fixed cubic shape and were not able to capture the flow characteristics. However, FEA can also be used to model the melt flow and material deposition [21,22]. In such cases, adaptive meshing must be used to modify the old elements progressively and generate new elements during transient simulation. This methodology has been used for small-scale desktop material-extrusion printing. It requires more computational resources, because the material flow is fully modeled and the melt geometry and free surface configuration during deposition is an unknown which has to be determined at each solution step. In this work, we present the results of this approach applied to large-scale material-extrusion AM. To validate the simulation results, an infrared camera was used to monitor the temperature history during material deposition.

**Figure 1:**
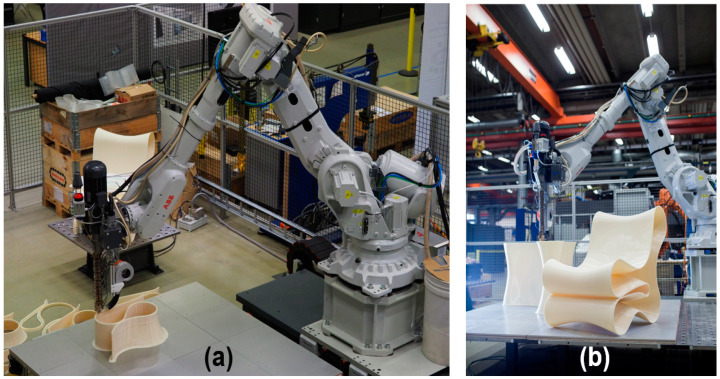
(**a**) IRBAM system setup printing a full-size chair from ABS. (**b**) Photograph of the printed chair.

The printing system used in this research is referred to as industrial robot-based additive manufacturing (IRBAM, Figure 1), developed at Research Institutes of Sweden (RISE) to fabricate various structures from acrylonitrile butadiene styrene (ABS) and polypropylene pure polymer as well as polymers reinforced with glass fiber (GF) and cellulose fibers. The system can deposit melted material at a rate of 6 kg/hour with a 6 × 2 × 2 m build volume. A transient two-dimensional FEA model was developed to predict the temperature rate and gradient during printing process. An infrared imaging camera was also used to monitor temperature evolution during material deposition. The model predictions were in good agreement with the experimental data of measured temperature. With further developments, this model can provide a basis for effective selection of printing parameters.

## 2. Printing Failures

Depending on parameters such as part geometry, printing material, melt temperature, cooling rate, and printing strategy, structures fabricated using large-scale AM may exhibit various failure modes, including cracking, distortion, and warpage. Several examples of printing failures are presented in this section to demonstrate the importance of process simulation.

**Figure 2:**
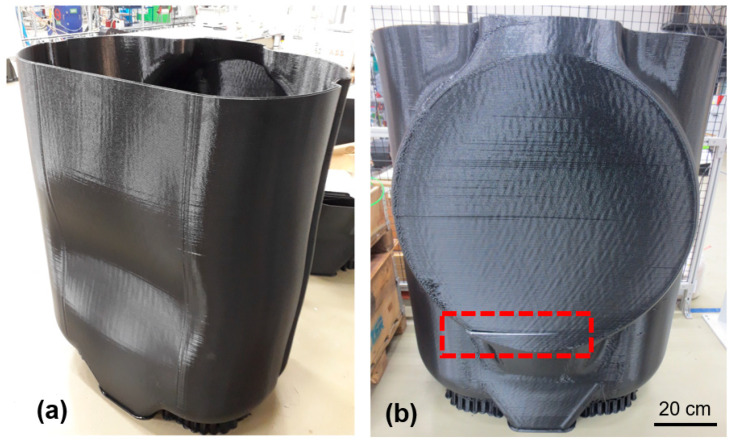
Manufacturing defects in a fuel tank printed from pure ABS using IRBAM. (**a**) Warpage and buckling in front side. (**b**) Cracking in the back side.

**Figure 3:**
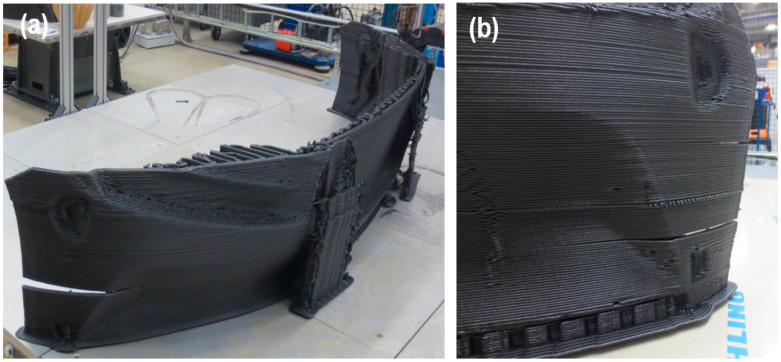
**(a**) Cracking and delamination in a vehicle bumper printed from ABS+15%GF. (**b**) Enlarged view of cracking locations.

Once cooled from deposition temperature to ambient conditions, printed polymer undergoes significant shrinkage. This may induce large stresses at the boundaries of layers. Figure 2 shows warpage and cracking in a fuel tank with a height of 1.5 m printed using ABS. These defects prevent engineering application of this part, despite the great time and material expended to manufacture it. Substantial deformation and buckling were observed on the front side, while a crack propagated on the back side. Additionally, Figure 3 demonstrates delamination in an automotive bumper printed from ABS+15%GF. Substantial delamination and cracks as long as 30 cm were observed on both sides of the bumper.

**Figure 4:**
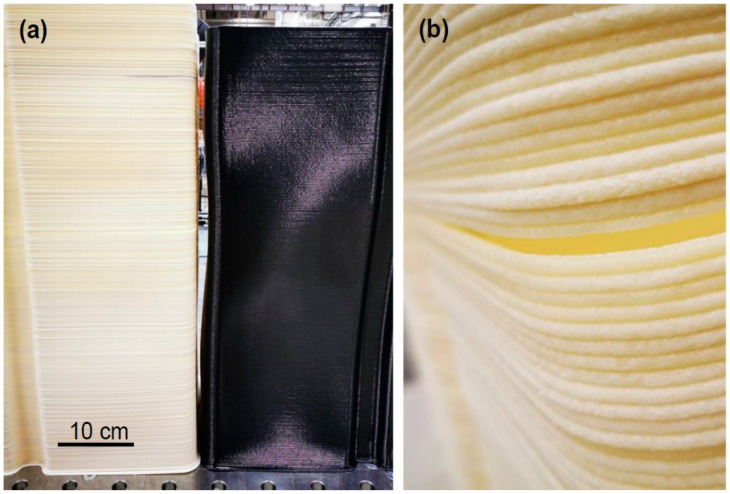
(**a**) Photograph of two straight boxes printed from ABS+15%GF (**left**) and pure ABS (**right**). (**b**) Enlarged view of delamination at the corner of the ABS+15%GF box.

Warpage and delamination may be observed in both polymer and composite (polymer + fiber) parts, although they may exhibit these failure modes to a different extent. To show this in detail, two boxes with identical initial geometries (in CAD) were printed from ABS+15%GF and pure ABS, as shown in Figure 4. In the case of the pure ABS material, substantial shrinkage and buckling was observed. When comparing the total height of the printed part, the pure ABS material resulted much greater deviation in comparison to the original CAD file. The deviation between the ABS and ABS+15GF parts compared to the CAD file was ~4.0% and ~0.5%, respectively. On the other hand, the pure ABS part showed a better surface finish, as it underwent a uniform shrinkage in all directions. As shown in Figure 4b, the ABS+15%GF part lacked a smooth surface finish owing to the nonuniform shrinkage of the deposited layers with chopped glass fibers distributed in different orientations. The orientation of reinforcing fibers influences the shrinkage significantly [23,24]. Furthermore, glass fibers accumulated at the interface between layers created stress concentration points, prevented effective bonding of the successive layers, and caused cracking and delamination. In addition, compared with pure ABS parts, it was easier to separate samples printed with ABS+15%GF from the build plate when the build plate temperature was lowered to room temperature. Overall, addition of fibers lowered the bonding strength between the printed layers as well as between the printed part and the build plate.

Addition of glass fibers enhances the mechanical performance and tensile strength of the part parallel to the printing direction, but it lowers the strength in the transverse direction (normal to build plate). Delamination in the fiber-reinforced parts (Figure 4b) showed that the transverse strength in these parts was even lower than that of the unreinforced pure polymer. Reduction of transverse mechanical properties can limit the structural applications of parts printed from fiber-reinforced polymer. This behavior can be compared to continuous (long)-fiber-reinforced composite laminates with a transverse tensile strength at the same order as or lower than that of the pure polymer [25,26,27]. Furthermore, reinforced fibers decrease the coefficient of thermal expansion and increase the thermal conductivity. Higher thermal conductivity in composite parts accelerates heat dissipation and prevents the large temperature gradients responsible for warpage of pure polymer parts.

## 3. Process Simulation

**Figure 5:**
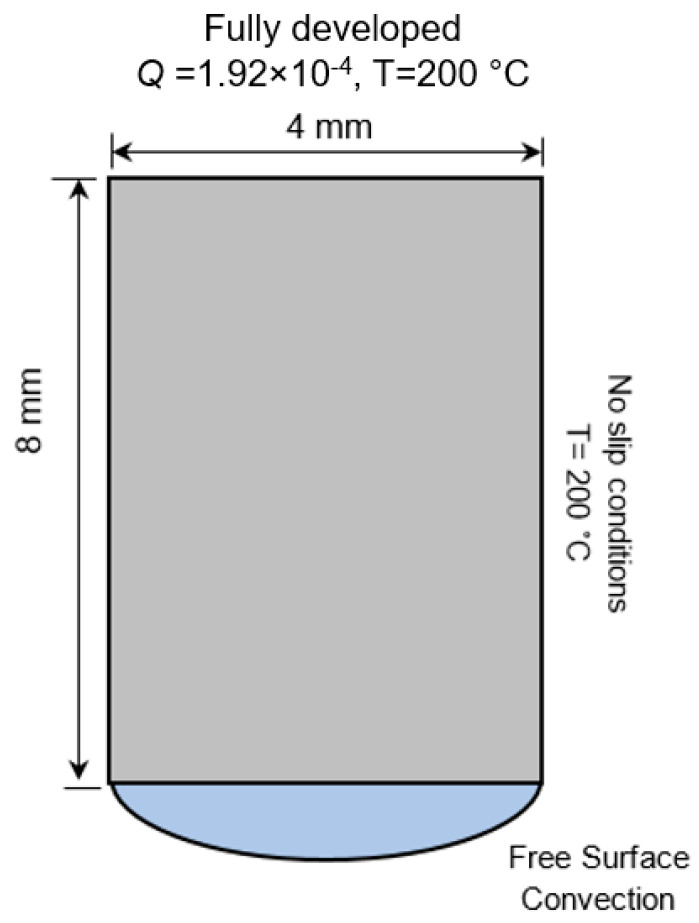
Schematic representation of thermal and flow boundary conditions applied to the model (not to scale).

**Figure 6:**
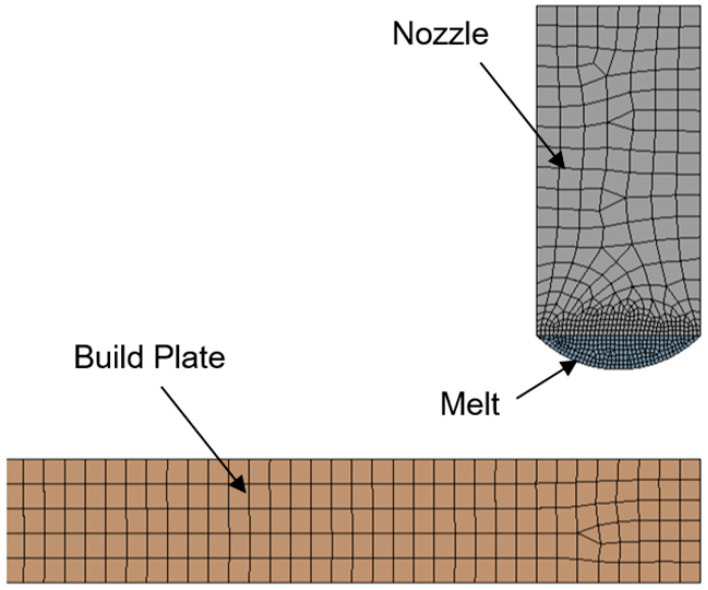
Initial element size distribution of the build plate, the nozzle, and the melt.

ANSYS Polyflow v19, originally developed to simulate the injection molding and extrusion processes, was adopted to simulate material deposition using large-scale extrusion-based AM. Sufficient flow and thermal boundary conditions were defined so that the model could accurately predict the melt temperature distribution in transient thermal-flow analysis (Figure 5). On the nozzle wall, no-slip conditions were prescribed, meaning the melt was attached to the wall and there was no relative displacement between the melt and the wall on the wall surface. The melt and build plate initial temperatures were set to 200 °C and 120 °C, respectively. A free surface convection condition was applied to the melt free surface after extrusion from the nozzle to simulate heat dissipation to the ambient environment. The ABS material properties used for the model are reported in Table 1. A power-law rheological model was used to describe the polymer viscosity in terms of temperature and strain rate [19]. Figure 6 shows the initial meshing of the nozzle, the melt, and the build plate.

In the transient thermal-flow simulation, an adaptive meshing algorithm was implemented in order to create new elements or modify the existing elements during the melt deposition as well as during the melt contact with the build plate or the previous layers. This means that the criteria used to assess the mesh quality during simulation may change over time, so that a mesh that was initially acceptable at the beginning of the simulation may be no longer adequate and rejected in the subsequent steps of the simulation. The adaptive meshing was integrated into the simulation setup to solve these issues. It regularly evaluates the mesh quality during transient simulation to detect highly distorted elements and replace them with higher-quality elements. For large-scale printing simulation, adaptive meshing is the most critical step. Almost 90% of the effort related to the process simulation in this work was devoted to adaptive meshing.

The size of the new elements after remeshing in ANSYS Polyflow was determined using two parameters: the quality parameter *T_quality_* and the size parameter *S*. The quality parameter *T_quality_* is a dimensionless parameter, and its default value is 0.8. It is a global measure which controls the geometric features of each element, such as angles between edges and aspect ratio. The value of the element quality can vary between 0 and 1. A quality value of 1 means that the element has maintained its original shape and is not distorted or deformed, while a value of 0 means that the element is completely distorted and has become flat.

**Figure 7:**
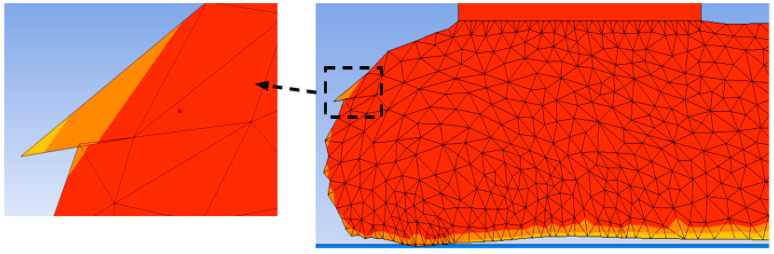
Element distortion in deposited melt after remeshing with the element quality value of *T_quality_* = 0.5.

Appropriate selection of *T_quality_* prevents the formation of distorted elements. If *T_quality_* is not large enough, distorted elements may form during the solution procedure and abort the simulation. An example is shown in Figure 7, where *T_quality_* = 0.5 generated distorted elements near the melt edge and caused the simulation to converge. It should be noted that ANSYS Polyflow only creates triangular elements for adaptive meshing. On the other hand, large values of *T_quality_* may create very fine elements and significantly increase the computation time. For instance, the simulation time with *T_quality_* = 0.7 for deposition of a single layer (10 cm long and 4 mm thick) was around one week when employing a 16-core processor and 64 GB of RAM. The default value of this parameter was 0.8, which was not suitable for large-scale printing simulation and significantly increased the simulation cost. To lower the computation resources needed in this study, the element quality parameter was set as *T_quality_* = 0.6. Overall, the computation time for large-scale extrusion AM is at least three orders of magnitude longer than that for small-scale AM.

The size parameter *S* has units of length, and its original value is compatible with the typical size of the initial mesh. If an element quality is below the specified value of *T_quality_* or its size is not within the range [*S*/2, 2*S*], it will be selected for adaptive meshing and refined or replaced with new elements. In this study, the element size parameter was set as *S* = 0.3 × 10^−3^ [28].

Adaptive meshing is also activated for large variations of temperature and velocity fields within the melt elements. If the field variation within an element is more than 50%, the element is refined. On the other hand, if the field variation within an element is less than 10%, the element is coarsened.

**Figure 8:**
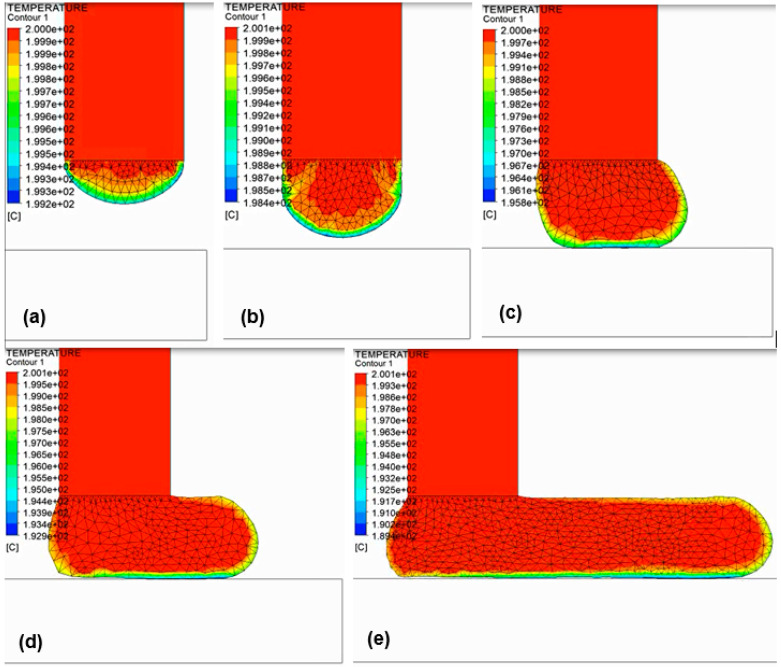
Snapshots of temperature contours during material deposition in large-scale AM. To account for the increase in material volume during extrusion, an adaptive remeshing scheme refines elements that have deformed significantly and become too stretched on the melt free surface.

**Figure 9:**
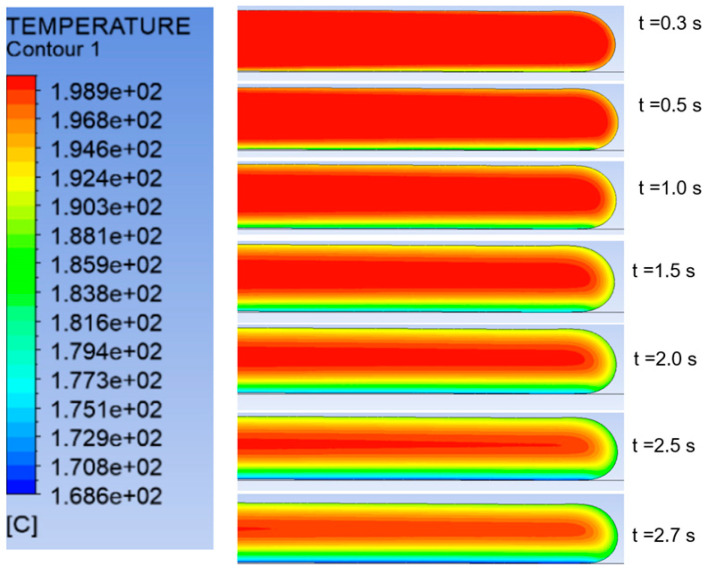
Simulation prediction of temperature change with time for a single 4 mm thick ABS layer after deposition.

A penalty technique is used to detect contact between the melt and the build plate. At the contact point, the condition that the melt velocity must be equal to the build plate velocity is enforced with a penalty coefficient, which was set to 1 × 10^16^ in the present simulation. If the penalty coefficient is not large enough, the melt elements will penetrate into the build plate elements and the contact detection will be inaccurate. Moreover, adaptive meshing is used to create new elements upon contact detection between the melt and the build plate. If the penetration of a point of the melt into the build plate is greater than the penetration accuracy, which was set to 1e-4 in this study, the current solution will be stopped and then repeated with a smaller time step. After contact occurs, the thermal boundary condition changes and the heat transfer between the melt and build plate is obtained according to the following equation:

*Q* = *α* (*T* − *T*_0_) (1)

where the coefficient *α* is the physical equivalence of a convection coefficient and captures the interfacial thermal resistance effects. Figure 8 depicts the temperature distribution history during deposition of a 4 mm thick layer of ABS. Mesh refinement is used to create new elements during melt deposition, as well as when melt contacts the build plate. Figure 9 shows the temperature change of the deposited layer with time.

## 4. Experimental Verification

**Figure 10:**
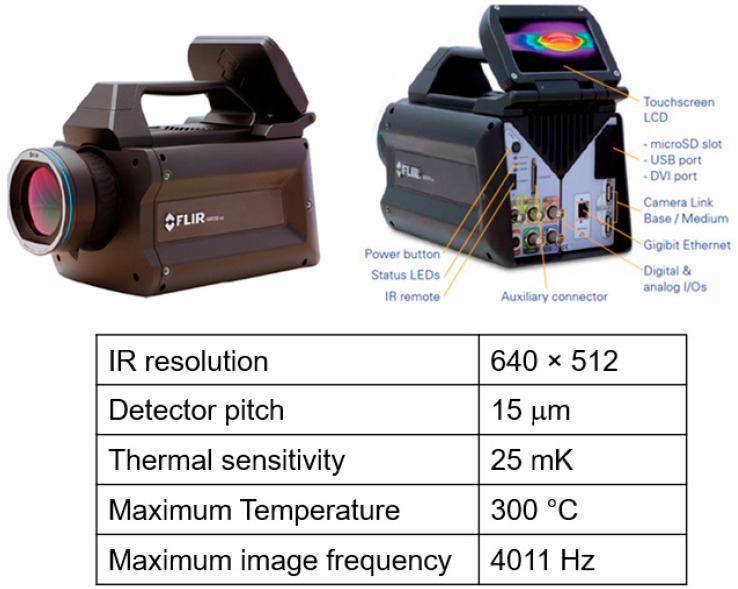
FLIR 6540sc infrared camera used to monitor temperature profile of the printed parts. The table summarizes the camera specifications [29].

**Figure 11:**
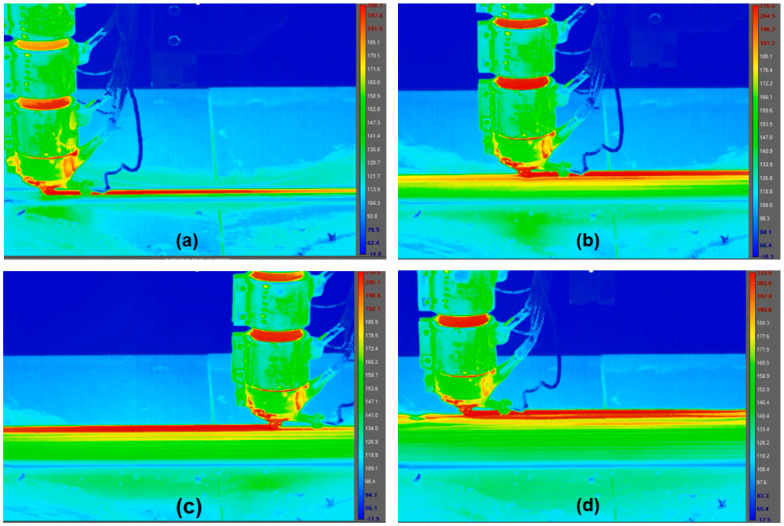
Snapshots of temperature profile recorded by the IR camera. A 700 mm long, 8 mm wide wall was printed with ABS.

The temperature history of a printed structure was recorded experimentally to validate the simulation predictions. A FLIR 6540sc infrared camera (FLIR Systems, Wilsonville, OR, USA) with an accuracy of ±1 °C or ±1% of the reading was used for temperature measurement (Figure 10). The IR camera was used to monitor the temperature profile during fabrication of a 700 mm long, 8 mm wide wall. The layer thickness was 4 mm. The camera was placed approximately 2.1 m from the central portion of the wall to capture the entire height of the wall. During the printing process, the camera captured a video at a frame rate of 12 Hz. Research IR software was used to analyze the video and extract the temperature data for comparison with the model. Snapshots of temperature measured by the IR camera are presented in Figure 11.

**Figure 12:**
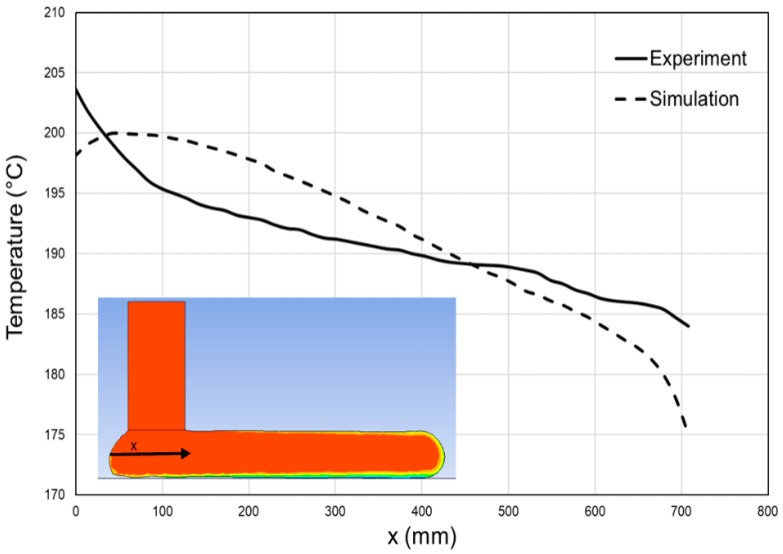
Model predictions and experimental data of temperatures along the mid-axis of a single layer deposited. The inset shows the origin of the *x* axis, located right underneath the nozzle along the middle plane of the deposited layer.

**Figure 13:**
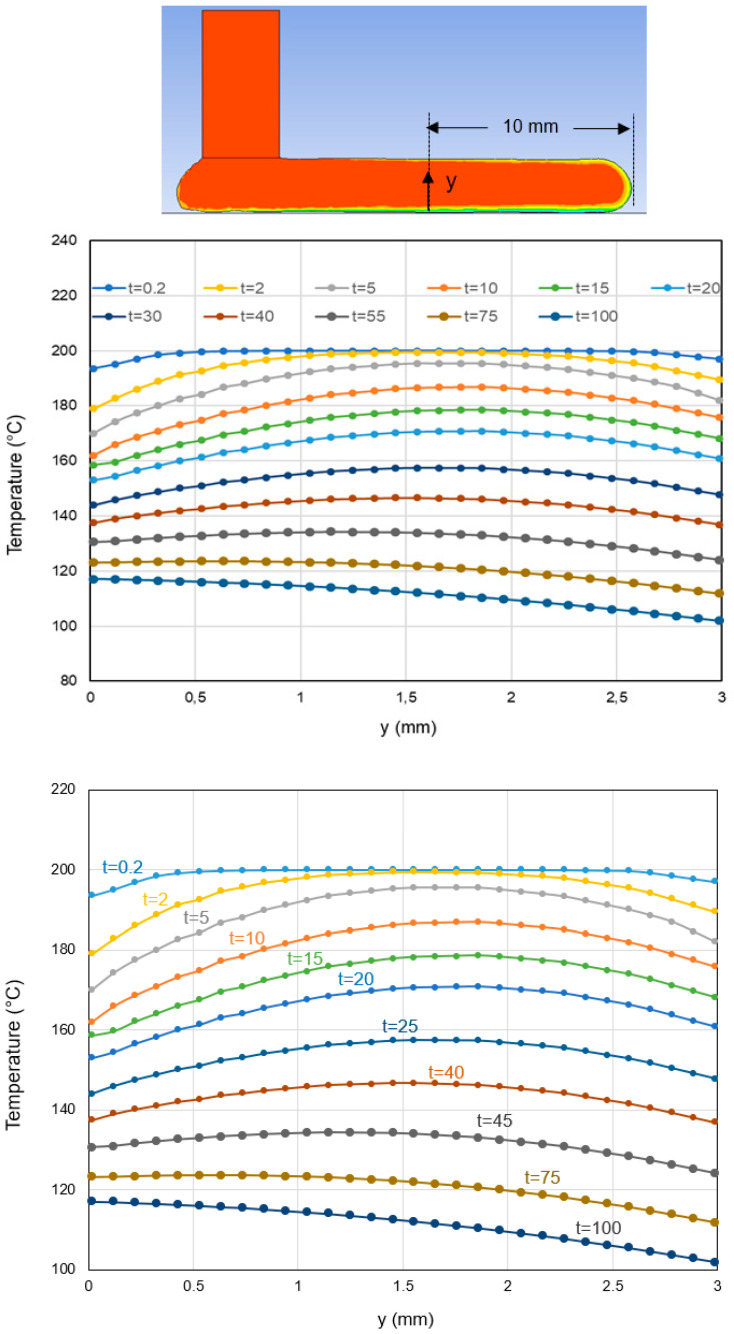
Simulation perdition of temperature change with time along the thickness of a single 4 mm thick ABS layer.

To validate the modeling approach developed in the previous sections, the model predictions of temperature distribution along the middle-plane of a single layer were compared with the measured temperature data (Figure 12). The model results agreed reasonably well with the measured data. A major difference is that the temperature reduction of the melt after deposition for *x* > 50 mm was slower in the experiment than in the model, probably due to inaccurate convection coefficients assigned to the melt free surface. The verified model enabled prediction of the temperature along different paths, i.e., through the thickness of the deposited layer, as shown in Figure 13. It showed that almost 40 s after deposition of a 4 mm thick layer, the temperature became uniform through the layer thickness. In order to capture stress and deformation evolution properly, polymer thermoviscoelastic behavior as well as shrinkage and crystallization kinetics should be characterized experimentally and incorporated into the model.

In large-scale printing, the thickness of each deposited melt layer is massive. If the material deposition is fast enough, the time will be insufficient for the temperature to dissipate and cool down. The question may then arise of whether it is possible to accurately simulate the temperature variation in a single layer by only using a transient thermal model, instead of a transient thermal-flow model. This is to avoid complicated and time-consuming adaptive meshing schemes required for flow analysis. To investigate this hypothesis, the deposition of one layer was simulated using both thermal and thermal-flow models. In the thermal model, it was assumed that the melt layer had already been deposited, and all the points started to cool and exchange heat with the ambient surroundings and the build platform simultaneously. Thus, the thermal model used a fixed mesh, which does not change during heat transfer. This reduced the simulation time by three orders of magnitude. On the other hand, in the more realistic thermal-flow model, deposition of the layer from the beginning was modeled, and the required remeshing schemes needed to model the material deposition and the melt contact with the build plate were considered.

**Figure 14:**
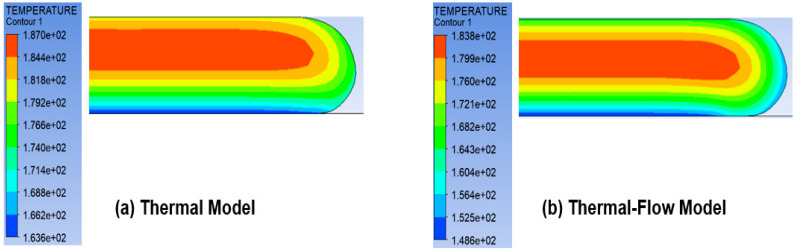
Comparison of temperature distribution for thermal and thermal-flow simulations. The temperature contours were obtained 10 s after deposition of a 3 mm thick layer started.

Figure 14 compares the outcome of the two models. The difference of maximum and minimum temperatures was around 3 °C and 15 °C, respectively. The large difference in minimum temperature shows that the layer had already started cooling during deposition, and therefore the flow of the material must be modeled and the thermal model may yield inaccurate results.

Thermal stresses can lead to significant residual stresses and deformations. The procedure established here for thermal simulation can be incorporated into a multiphysics simulation to obtain the stress distribution. This may enable accurate prediction of failure locations. The failure can then be avoided by modifying printing parameters such as nozzle diameter, printing path, or material deposition rate. The model presented here enables thermal simulation for a variety of printing variables.

## 5. Conclusions

Different failure modes observed in polymer and composite parts fabricated via large-scale extrusion AM were reported. Reinforcing the polymer matrix with fibers can change the dominant failure mode from warpage to delamination. The extensive failure in the printed parts shows the importance of process simulation for damage prediction. As a first step, thermal simulation was conducted to determine the temperature history. We used a numerical model to determine the temperature change for layers printed using a large-scale robotic printing system. An adaptive meshing scheme was applied to the melt area to generate new elements during material deposition in the transient analysis. Element size and shape in the nozzle and build plate area was assumed to be constant. For a 4 mm thick ABS layer, the temperature history was obtained over 100 s after deposition. It was shown that almost 40 s after deposition of a 4 mm thick layer, the temperature became uniform throughout the layer thickness. An infrared camera was used to monitor temperature history while printing ABS. The agreement of numerical and experimental data verified the simulation process. The model can be further extended for stress analysis in printed parts.

## Figures and Tables

**Table 1:** ABS Material Properties [19].

Glass transition temperature (*T_g_*)	105.0 °C
Coefficient of thermal expansion (CTE)	7.38 × 10^−5^ m/(m·°C)
Specific heat (*c*)	1423.51 J/(kg·°C)
Thermal conductivity (*k*)	0.17 W/(m·°C)
Density (*ρ*)	1040 kg/m_3_

## Data Availability

The data presented in this study are available on request from the corresponding author.
